# Inhibition of radiation induced migration of human head and neck squamous cell carcinoma cells by blocking of EGF receptor pathways

**DOI:** 10.1186/1471-2407-11-388

**Published:** 2011-09-06

**Authors:** Anja C Pickhard, Johanna Margraf, Andreas Knopf, Thomas Stark, Guido Piontek, Carolin Beck, Anne-Laure Boulesteix, Elias Q Scherer, Steffi Pigorsch, Jürgen Schlegel, Wolfgang Arnold, Rudolf Reiter

**Affiliations:** 1 Department of Otolaryngology Head and Neck Surgery, Technical University of Munich, Ismaninger Straße 22, 81675 Munich, Germany; 2 Institute of Pathology, Section of Neuropathology, Technical University of Munich, Ismaninger Straße 22, 81675 Munich, Germany; 3 Institute for Statistics, Ludwig-Maximilians-University Munich, Ludwigstraße 33, 80539 Munich, Germany; 4 Department of Radiotherapy, Technical University of Munich, Ismaninger Straße 22, 81675 Munich, Germany; 5 Department of Otolaryngology Head and Neck Surgery, Section of Phoniatrics and Pedaudiology, University of Ulm, Prittwitzstr. 43, 89070 Ulm, Germany

## Abstract

**Background:**

Recently it has been shown that radiation induces migration of glioma cells and facilitates a further spread of tumor cells locally and systemically. The aim of this study was to evaluate whether radiotherapy induces migration in head and neck squamous cell carcinoma (HNSCC). A further aim was to investigate the effects of blocking the epidermal growth factor receptor (EGFR) and its downstream pathways (Raf/MEK/ERK, PI3K/Akt) on tumor cell migration in vitro.

**Methods:**

Migration of tumor cells was assessed via a wound healing assay and proliferation by a MTT colorimeritric assay using 3 HNSCC cell lines (BHY, CAL-27, HN). The cells were treated with increasing doses of irradiation (2 Gy, 5 Gy, 8 Gy) in the presence or absence of EGF, EGFR-antagonist (AG1478) or inhibitors of the downstream pathways PI3K (LY294002), mTOR (rapamycin) and MEK1 (PD98059). Biochemical activation of EGFR and the downstream markers Akt and ERK were examined by Western blot analysis.

**Results:**

In absence of stimulation or inhibition, increasing doses of irradiation induced a dose-dependent enhancement of migrating cells (p < 0.05 for the 3 HNSCC cell lines) and a decrease of cell proliferation (p < 0.05 for the 3 HNSCC cell lines). The inhibition of EGFR or the downstream pathways reduced cell migration significantly (almost all p < 0.05 for the 3 HNSCC cell lines). Stimulation of HNSCC cells with EGF caused a significant increase in migration (p < 0.05 for the 3 HNSCC cell lines). After irradiation alone a pronounced activation of EGFR was observed by Western blot analysis.

**Conclusion:**

Our results demonstrate that the EGFR is involved in radiation induced migration of HNSCC cells. Therefore EGFR or the downstream pathways might be a target for the treatment of HNSCC to improve the efficacy of radiotherapy.

## Background

Head and neck squamous cell carcinoma (HNSCC) is the sixth most common cancer worldwide [1]. In case of a primary radiotherapy patients get no surgery. Therefore radiation doses need to be higher than in those cases where the patient gets surgery and a postoperative adjuvant radiotherapy.

Anti-neoplastic properties of ionizing radiation are primarily related to DNA damage. This treatment is an established measure for HNSCC therapy [2,3]. Despite technological advances and increased radiation intensity only approximately half of the patients get cured [4]. The outcome of patients presenting more advanced stages is even poorer, with 5-year actuarial survival rates of about 30% [5]. These findings underscore the need to develop novel strategies in the management of patient with advanced HNSCC.

In the last decade significant progress has been made in the understanding of the molecular mechanisms that are responsible for human cancer development and progression. The epidermal growth factor receptor (EGFR), a member of the structurally related erbB family of tyrosine kinase receptors, has been implicated in cancer development and progression in a large number of tumors including HNSCC [6]. EGFR over-expression occurs early in the pathogenesis of HNSCC [7] and is associated with reduced relapse-free survival or poor overall survival time [8]. Also a new study shows, that EGFR protein levels strongly predict for patient outcome in HNSCC [9]. At a clinical level, inhibition of EGFR with monoclonal antibody showed therapeutic effects with better survival of patients when added to standard radiotherapy [10]. In advanced or metastatic tumors cetuximab plus chemotherapy had significant effects compared with chemotherapy alone on outcome of overall survival and progression-free survival [11].

Interestingly, in a glioma cell model it has been shown that sublethal irradiation promotes migration and invasion of tumor cells [12].

It has been shown on a molecular level that radiation induces an overexpression of EGFRs in many HNSCC [7,13,14]. Cassell et al. mentioned that inhibition of EGFR with a monoclonal antibody (cetuximab, Erbitux™), enhanced the development of more effective HNSCC treatments. But there is a need of a prospective identification of patients who would benefit from such a therapy [15]. Besides, a phase III randomised trial has shown that the combination of radiotherapy with the EGFR antibody cetuximab significantly improves overall survival at 5 years [16].

Molecular research has identified a host of new biological parameters with potential predictive utility. Oncogenes, tumor suppressor genes, cell-cycle control genes, apoptosis genes and angiogenesis genes have been extensively studied and correlated with radiation response [17,18].

Akt (protein kinase b) as a possible response modulator has recently fostered molecular strategies which employ blockade of the receptor to down-regulate tumor growth [19]. Besides, inhibition of Rhokinase or PI3 kinase decreases tumor growth and cisplatin resistance in HNSCC [20]. Also, expression levels of phosphorylated Akt and mTOR are higher in HNSCC than in non-cancer patients [21].

**Figure 1:**
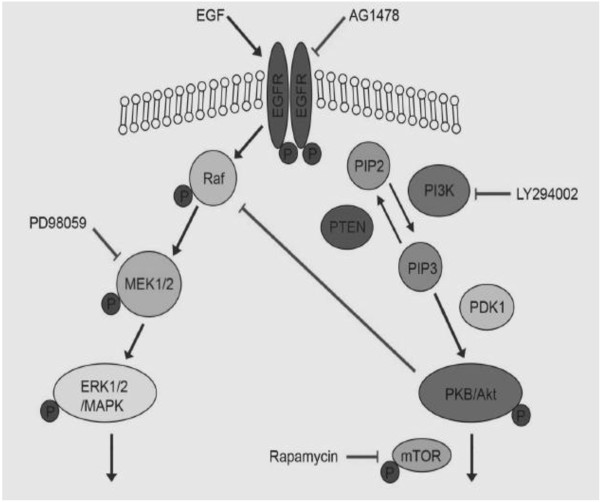
**Components of the EGFR pathways and their specific inhibitors**. EGFR (Epidermal growth factor receptor), PIP2, PIP3 (Phosphatidylinositol-4,5-bisphosphate (PIP2), Phosphatidylinositol 3,4,5-trisphosphate (PIP3), MAPK/ERK (mitogen activited proteinkinase), mTOR/FRAP (Mammalian target of rapamycin), PI3K (Phosphatidylinositol-3'-Kinase), PKB/Akt (Proteinkinase B), PTEN (Phosphatase and tensin homologue deleted on chromosome ten).

The PI3K dependent pathway and the ERK pathway are important pathways for tumor biology [22]. Raf/MEK/ERK connect mitogen signals [23], whereas the PI3K dependent activation of the Akt seems to be important for anti-apoptosis and migration [24,25] (Figure 1).

The purpose of our study was twofold: (1) to investigate radiation induced migration of the well established HNSSC cell lines (BHY, CAL-27 and HN) and (2) to investigate the possibility of inhibiting migration by blocking the EGF receptor pathways.

## Methods

### Cell culture and irradiation

The cell lines HN, BHY [26] and CAL-27 [27] were used (DSMZ, Braunschweig, Germany). Cells were grown in Dulbecco's modified Eagle medium (DMEM) or Roswell park memorial institute medium (RPMI 1640) (Invitrogen, Karlsruhe, Germany) containing 10% fetal calf serum, 2 mM glutamine, and 100 μg/ml penicillin/streptomycin and maintained at 37°C in an atmosphere of 5% CO_2 _grown to a 70-90% confluence.

Irradiation was performed at the Department of Radiotherapy (Technical University of Munich). Cells were X-irradiated with single doses of 2, 5 or 8 Gy with a Philips RT 100 (Philips, Amsterdam) operated at 300 kV with 1.4 mm copper half-value layer at a dose rate of approximately 1 Gy/min. The dose inhomogeneity was ± 2%. The sham-treated group (0 Gy, control) was subjected to the same protocol as exposed cells.

### Wound healing assay

**Figure 2:**
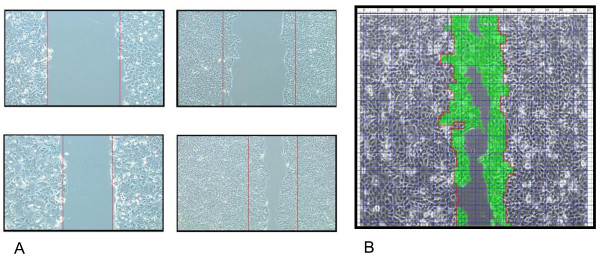
**A+B. Migrating cells of the wound healing assay**. (A) Migrating cells of the wound healing assay -/+ irradiation were presented as pictures (40 fold magnification, Axiovert 25, Carl Zeiss AG, Göttingen) 12 hours after irradiation. (B) The pictures were fused and the highlighted cells were counted.

Investigation of cell migration capability after irradiation treatment was performed by a modified wound healing assay, as described before [28]: Briefly, treated and untreated cells were grown to confluent monolayers. Immediately before irradiation the inhibitors rapamycin (100 nM) (Biomol, Hamburg, Germany), LY294002 (50 μM) (Calbiochem, Darmstadt, Germany), PD98059 (50 μM) (Biomol, Hamburg, Germany), tyrphostin AG1478 (10 μM) (Merck, Darmstadt, Germany) or the epidermal growth factor (EGF) (10 ng/ml) (Upstate, Billerica, USA) were separately added to the medium. After that, the monolayers were wounded by scratching the surface as uniformly as possible with a 200 μl pipette tip (Sarstedt AG & Co., Nümbrecht, Germany). After irradiation cells were cultivated for another 12 hrs. This initial wounding (0 hr) and the movement of the cells in the scratched area were photographically monitored under an inverted light microscope (field of view by a 40 fold magnification - Axiovert 25, Carl Zeiss AG, Göttingen, Germany, equipped with an Olympus SC 35 Camera, Volketswil, Switzerland). Migrating cells were counted 12 hours after irradiation (Figure 2). These time points were chosen because in former experiments all cells were grown to confluence after 36 hours (data not shown).

### Modified Boyden chamber

To confirm the results of the scratch test we analyzed the migration by a modified Boyden chamber. Cells were given to a transwell permeable polycarbonate membrane with a pore size of 0.8 μm (Corning Incorporated, New York, USA). Inhibitors were added in the medium above and under the membrane then irradiation were done. 12 hrs later cells above the membrane were removed by a cotton drill, and fixed with DAPI. Thereafter cells were counted under a microscope. These experiments were performed to show consistent and comparable results of radiation induced migration.

### Proliferation assessment

The MTT [3-(4,5-dimethylthiazol-2-yl)-2,5-diphenyltetrazolium bromide] assay (Roche Diagnostics, Penzberg, Germany) was used to assess cell proliferation, as previously described [29]. Briefly, cells were plated on 96-well plates at a concentration of 1000 cells/well. The above-mentioned inhibitors were added 12 hours prior to irradiation. After incubation, and 12, 24 and 72 hours after irradiation, 10 μl of MTT solution was added to each well for four hours (37°C). Subsequently, 100 μl of dimethylsulfoxide was added to each well, yielding purple solution. The optical density was measured at 590 nm using an ELISA reader (ASYS Hitech, Eugendorf, Germany) and ratios in relation to controls were made. All experiments were performed eight times (n = 8).

### Immunoblot analysis

Immunoblot analysis was performed to determine EGFR expression including its downstream proteins ERK and Akt. 12 hours after irradiation, cells were harvested in lysis buffer (Cell Lysis Buffer, New England Biolabs, Ipswich, USA) at 4°C. Lysates were centrifuged (10000 rpm) for 15 minutes at 4°C to remove insoluble components. Protein content was quantified by the Bio-Rad Dc protein assay (Bio Rad, Hercules, USA). Equal amounts of protein were separated on SDS-PAGE 10% or 12.5% gels. Proteins were transferred to Immobilon-P PVDF membrane (Millipore, Billerica, USA). The membranes were blocked with 5% nonfat dry milk in Tris-buffered saline containing 0,1% Tween 20 (TBST) and afterwards incubated with primary antibody in 5% nonfat dry milk in TBST, followed by secondary antibody linked to rabbitradish peroxidase diluted in 5% nonfat dry milk in TBST. ECL Detection System for Western blot Analysis (Amersham, Freiburg, Germany) was used according to the manufacturer's instructions. The Imager SRX-101° (Konica Minolta, Langenhagen, Germany) was used to detect bands of appropriate sizes. The following antibodies were used: phospho-EGFR (Tyr1068), phospho-Akt (Ser473), PKB/Akt, phospho-p44/42 ERK (Thr202/Tyr204), p44/42 ERK, phospho-Raf (Ser259), phospho-MEK1/2, and MEK1/2. All antibodies were obtained from Cell Signaling Technology, (Boston, USA) and used at a dilution of 1:1000.

### Data and statistical analysis

For the investigation of cell migration, a two-factorial design was considered with the factors "treatment" (control, EGF, LY294002, PD98059, rapamycin, AG1478) and radiation dose (ranging from 0 Gy to 8 Gy). The whole analysis was repeated n = 9 times. Cell proliferation was investigated for all six treatments, for doses 0 Gy and 8 Gy only. For each dose and each group, the sample size was n = 8. In the whole study, the cells were randomly assigned to the treatment groups and radiation doses.

Radiation induced migration was assessed in a linear regression model were migration was set as dependent variable and the radiation dose as the metric predictor. The potential dose dependent inhibition or enhancement of migration through stimulation was investigated based on a generalized least squares model fitted with the R function 'gls' with the migration as dependent variable. The predictors were: the radiation dose (metric predictor, coefficient β_rad_), the treatment (categorical predictor with coefficients β_EGF_, β_AG_, etc and controls as reference category), and their interactions (coefficients β_rad.EGF_, β_rad.AG_, etc). A uniform correlation structure was assumed within each of the n = 9 experiments, corresponding to a linear mixed model with a random forest for each experiment. Residual analysis showed that this model can reasonably be applied to the data at hand. Additionally, linear hypotheses tests were performed in the above GLS model to test the effect of the radiation dose in the presence of treatment (tested hypotheses: β_rad_+β_rad.EGF _= 0, β_rad_+β_rad.AG _= 0, etc). Confidence intervals for the estimated coefficients were calculated, and all hypotheses were tested based on the Wald test. Separate analyses were conducted for the three cell lines BHY, CAL-27 and HN for the time point 12 hours. The t-test was used to compare proliferation in two conditions. Confidence intervals for the difference of means were calculated. All statistical analyses were performed using the R statistical software http://www.r-project.org, version 2.6.1.

## Results

### Blocking of EGFR decreased radiation induced migration

**Figure 3:**
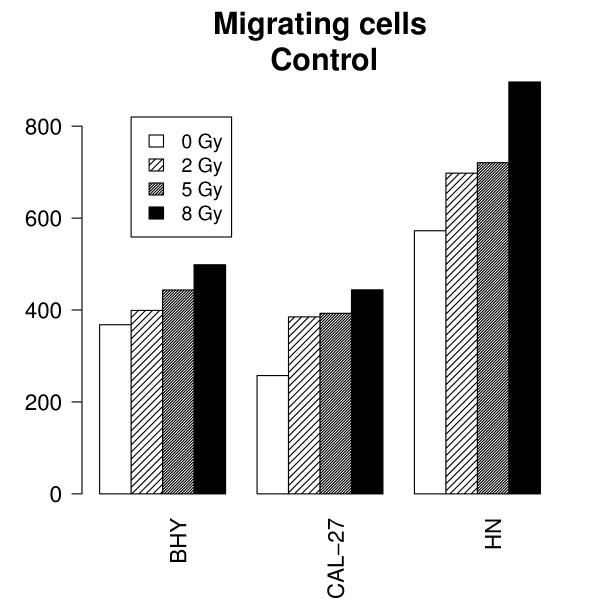
**Radiation induced migration**. The number of migrating cells after irradiation with 0, 2, 5, and 8 Gy were determined by the wound healing assay for the HNSCC cell lines BHY, CAL-27 and HN. Cells were grown to confluent monolayers, wounded by scratching the surface as uniformly as possible with a pipette tip and afterwards irradiated. The number of migrated tumor cells in the wound was determined. The means and standard errors of 9 tests per time point and dose were calculated after 12 hrs. An increased time dependent migration of tumor cells after irradiation was observed after treatment compared to the control group of not irradiated cells (p < 0.009).

The cultured cell lines BHY, CAL-27 and HN were irradiated with 2, 5 and 8 Gy and monitored during 12 hours. The means and standard errors of nine tests per time point and dose were calculated for each cell line. Radiation induced a significant dose dependent migration of tumor cells after irradiation compared to the control group (BHY: β_rad _= 16, CI:[6;26], p = 0.003, CAL-27: β_rad _= 20, CI:[10;30], p < 0.001, HN: β_rad _= 37, CI:[22;51], p < 0.001) (Figure 3).

The findings of the wound healing assay were consistent with the results of the modified Boyden chamber where a radiation induced migration was also observed.

**Figure 4:**
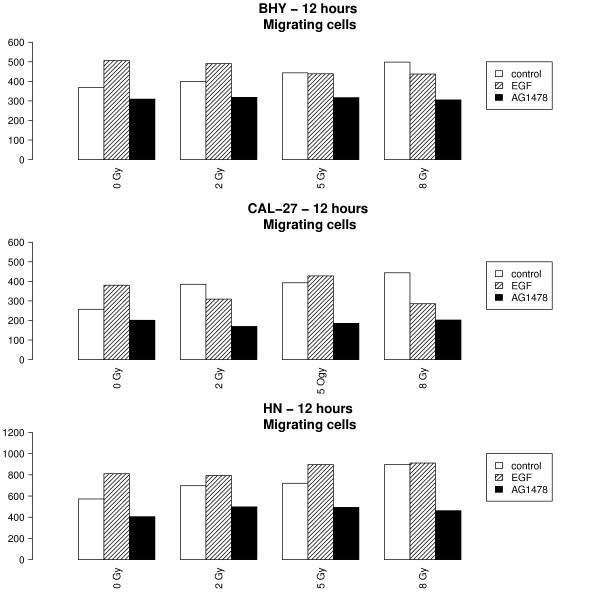
**AG1478 inhibits radiation induced migration**. Migration ability of the 3 different HNSCC cell lines after stimulation with EGF and blocking of the EGFR with AG1478 was determined by the wound healing assay. The means of 9 tests per dosage were calculated after 12 hrs. The increase of migration with increasing radiation dosis was significantly less pronounced after stimulation with EGF and after blocking of EGFR with AG1478 than in control cells.

The stimulation of not irradiated (0 Gy) cells with EGF lead to a significant increase of migration in all cell lines (tested hypothesis β_EGF _= 0, BHY: β_EGF _= 137 CI:[66;209], p < 0.001, CAL-27: β_EGF _= 79, CI:[1;156], p = 0.048, HN: β_EGF _= 211, CI:[113;308], p < 0.001). In contrast, the EGFR inhibitor AG1478 significantly decreased migration (Figure 4).

The radiation-induced increase of migration was significantly less pronounced after stimulation with EGF (tested hypothesis β_rad.EGF _= 0, BHY: β_rad.EGF _= -26, CI:[-41;-11], p < 0.001, CAL-27: β_rad.EGF _= -26, CI:[-42,-10], p = 0.002, HN: β_rad.EGF _= -21, CI:[-41,-1;], p = 0.042) as well as after inhibition with AG1478 (tested hypothesis β_rad.AG _= 0, BHY: β_rad.AG _= -17, CI:[-32;-2], p = 0.028, CAL-27: β_rad.AG _= -19, CI:[-35;-3], p = 0.021, HN: β_rad.AG _= -31, CI:[-52,-11], p = 0.003) than in control cells (Figure 3). More precisely, migration did not increase significantly with radiation dose in the cells stimulated with EGF or inhibited with AG1478 (tested hypotheses: β_rad_+β_rad.EGF _= 0, β_rad_+β_rad.AG _= 0, p > 0.05), in contrast to what happens in control cells.

### Radiation-induced migration can be blocked by inhibition of EGFR downstream pathways

**Figure 5:**
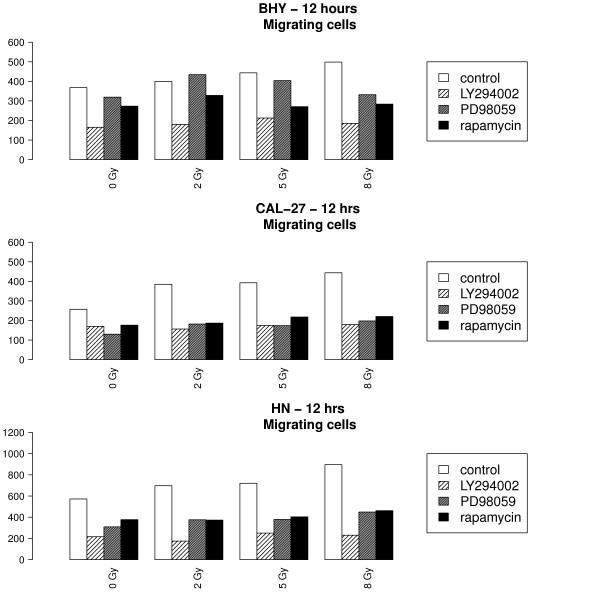
**Migration ability of the HNSCC cell lines after blocking of the downstream pathways of the EGFR**: PI3K by LY294002 (LY), mTOR by rapamycin (RA) and MEK1 by PD98059 (PD) after irradiation with 0, 2, 5 and 8 Gy after 12 hours. Measurements were made in 9 experiments. The effect of radiation on migration was significantly reduced by inhibitors of the downstream pathways of EGFR. (A cell line BHY, B cell line CAL-27, C cell line HN)

Additionally to the above mentioned inhibition of the EGF receptor, we blocked the downstream pathways of EGFR: PI3K by LY294002 (30 minutes before radiation), mTOR by rapamycin (1 hour before radiation) and MEK1 by PD98059 (30 minutes before radiation). A significant negative interaction between irradiation and the inhibitors was seen in the HN cell line after 12 hours. These findings indicate that the radiation-induced migration of tumor cells was decreased significantly by downstream inhibitors of the EGFR (Table 1). Migration was most effectively decreased by blocking of PI3K (LY294002) (Figure 5, Table 1).

**Table 1:** p-values.

time [hrs]	12						24					
**treatment**	**LY**	**PD**	**RA**	**LY+**	**PD+**	**RA+**	**LY**	**PD**	**RA**	**LY+**	**PD+**	**RA+**

**BHY**	< 0.001	0.781	0.050	0.089	0.023	0.021	< 0.001	0.007	0.011	0.131	0.125	0.005

**CAL**	0.001	< 0.001	0.003	0.027	0.107	0.088	< 0.001	< 0.001	< 0.001	0.154	0.301	0.024

**HN**	< 0.001	< 0.001	< 0.001	0.002	0.041	0.014	< 0.001	< 0.001	< 0.001	0.001	0.003	< 0.001

The p-values are shown for the effect of blocking in controls (tested hypotheses: β_LY _= 0, β_PD _= 0, β_RA _= 0) and for the interaction (+) with radiation (tested hypotheses: β_rad.LY _= 0, β_rad.PD _= 0, β_rad.RA _= 0, etc). All interaction coefficients were negative, indicating that the effect of radiation on migration was weaker after inhibition. (LY294002 (LY), PD98059 (PD), rapamycin (RA))

After inhibition migration did not significantly increase with radiation dose (tested hypotheses: β_rad_+β_rad.LY _= 0, β_rad_+β_rad.Rapa _= 0, β_rad_+β_rad.PD _= 0, p > 0.05) in all 3 cell lines, in contrast to what happens in controls. Thus, inhibition seems to attenuate the influence of radiation on migration.

### Proliferation subsided by inhibition of the PI3K/Akt pathway

The strongest migration ability was observed at the dose of 8 Gy. Therefore we focused on studying the effects elicited at this radiation dose. We found a significant decrease of proliferation after radiation with 8 Gy after 72 hours (BHY: CI:[-0.19,-0.12], p < 0.001; CAL-27: CI:[-0.13,-0.05], p < 0.001; HN: CI:[-0.07,-0.01], p = 0.014). Stimulation with EGF showed no significant effect on proliferation (BHY: CI:[-0.11,0.06], p = 0.5; CAL-27: CI:[-0.09,-0.01], p = 0.02; HN: CI:[0.02,0.09], p = 0.005) without radiation, and no significant effect by simultaneously radiation with 8 Gy (BHY: CI:[-0.06,0.10], p = 0.57; CAL-27: CI:[-0.05,0.01], p = 0.19; HN: CI:[-0.05,0.005], p = 0.10). After EGF receptor blockade with AG1478 a significant decrease in proliferation was observed, compared to the control group (BHY: CI:[-0.27,-0.15],, CAL-27: CI:[-0.20,-0.14],, HN: CI:[-0.13,-0.07], p < 0.001).

**Figure 6:**
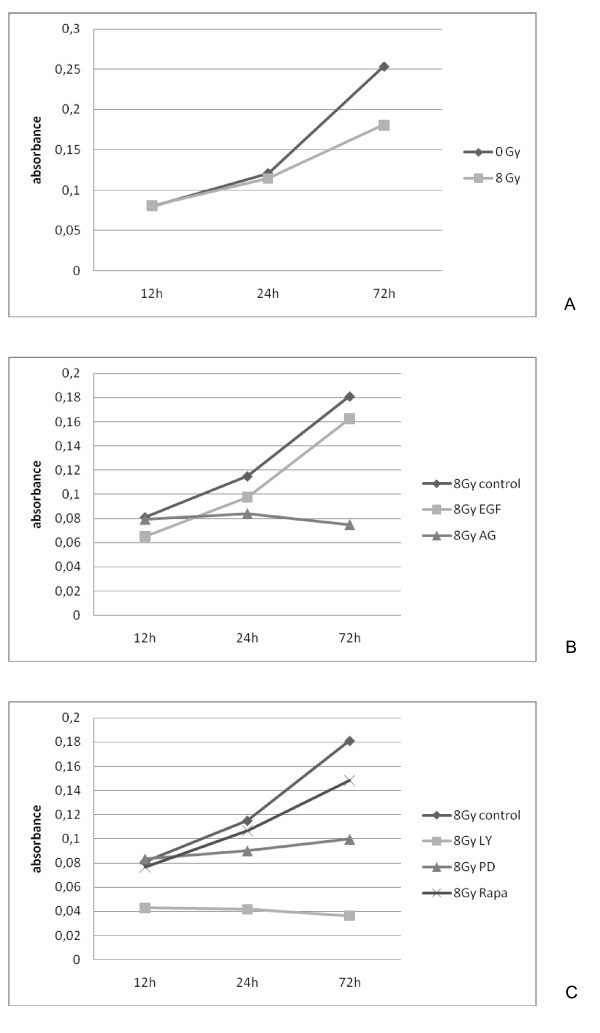
**A+B+C. Subsided proliferation after inhibition**. Proliferation was assessed by the MTT test 12 hrs, 24 hrs and 72 hrs after irradiation with 8 Gy or without irradiation (0 Gy). Proliferation subsided by radiation of the cell line CAL-27 and also by inhibition of the EGFR by AG1478 (AG) and after blocking of the downstream pathways of the EGFR: PI3K by LY294002 (LY), mTOR by rapamycin (Rapa) and MEK1 by PD98059 (PD), control.

Inhibition of MEK1 with PD98059 or inhibition of mTOR with rapamycin reduced significantly proliferation (BHY: CI:[-0.25,-0.12], CAL-27: CI:[-0.17,-0.10], HN: CI:[-0.15,-0.07], p < 0.001). Blocking of PI3K with LY294002 also reduced proliferation (BHY: CI:[-0.36,-0.31], CAL-27: CI:[-0.26,-0.20], HN: CI:[-0.24,-0.18], p < 0.001) (Figure 6).

### EGFR activation after irradiation was detected by Western blot analysis

**Figure 7:**
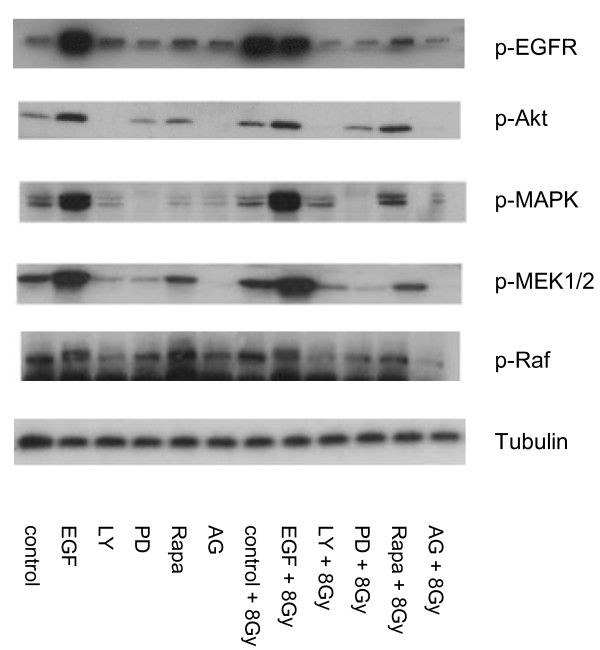
**pEGFR activation by irradiation**. The Western blot results were presented in figure form. Protein isolation was done 12 hrs after irradiation with 8 Gy. EGFR was stimulated by EGF and inhibited by AG1478 (AG) and the downstream pathways of EGFR: PI3K by LY294002 (LY), mTOR by rapamycin (Rapa) and MEK1 by PD98059 (PD), control. We find an up-regulation of phospho-EGFR after radiation.

Protein was isolated at 0 hours and 24 hours after radiation with 8 Gy. In all 3 cell lines we found a constitutive activation of Akt and ERK. The stimulation with EGF preceded an up-regulation of EGFR phosphorylation and a phosphorylation of the downstream pathways. Blockade of the EGFR by AG1478 provoked a down regulation of the receptor, the PI3K/Akt and the Raf/MEK/ERK pathways. This effect continued during 24 hours. After radiation an up regulation of the EGFR phosphorylation was observed. The western blot results are presented in Figure 7.

## Discussion

Survival rates of HNSCC patients have not improved during the last decades [1]. HNSCC cells distinguish through infiltrative growth in the surrounded area. This is the reason for locally advanced disease in over 40% of patients [30]. Often tumor location does not allow an in sano resection without severe impairment in functions like swallowing, speech or respiration. Therefore primary radiation therapy is an established therapy of inoperable HNSCC, but the prognosis is poor with five-year-cure rates rarely exceeding 50% [4,31]. Additionally, radiation combined with chemotherapy has been shown to be superior to radiotherapy alone. There are benefits in terms of survival and organ preservation [5]. Also new strategies like the combined-treatment with cisplatin and hyperfractionated radiation therapy maintained improved rates of locoregional control, distant metastasis-free survival, and cancer-specific survival [32]. But unfortunately, no criteria for response to the radiation therapy have been found.

In this study we could demonstrate for the first time a radiation induced migration of HNSCC cells like it is known for glioma cells [12,33]. Proliferating cells could make a misleading result in the wound healing assay, because they appear to imitate migration. However, this effect is not caused by proliferating cells, because it was shown in the MTT test that cell proliferation decreases, when cells are irradiated.

Our results showed that migration is increased by stimulation of the cells with EGF and by radiation treatment. The mechanism might be a radiation induced an autophosphorylation of the EGF receptor with an activation of the downstream pathways, previously observed [14,34]. Blockade of the EGFR by AG1478 that leads to a significant inhibition of migration might support this observation.

The EGFR plays an important role in tumor biology of HNSCC. In a systematic review, the EGFR signaling is associated with poor prognosis and response to therapy in cervical cancer patients primarily treated with chemoradiation [35]. Bonner et al. showed that the combination of radiotherapy and cetuximab improved the overall survival significantly [16]. Also Frampton found in the setting of locally advanced, unresectable disease, cetuximab plus radiation offers an alternative approach to the current standard of care, namely platinum-based chemotherapy plus radiotherapy and in recurrent and metastatic HNSCC, cetuximab plus platinum-based chemotherapy provides a first-line treatment of choice [36]. The reason for this might be a reduction in cell migration after blocking the EGFR in combination with radiation, as we observed. Recent studies give an account of Akt induced migration [37,38]. Therefore we focused on the EGFR downstream pathways Raf/MEK/ERK and PI3K/Akt and investigated whether a correlation with the radiation-induced migration existed. A relation between the PI3K/Akt signaling pathway and the migration was assumed, because inhibition of PI3K by LY294002 and blockade of mTOR by rapamycin involved a significant decrease of migrating cells. The same effect was seen after inhibition of MEK1 by PD98059. This was confirmed by our western blot results: after radiation we observed an up-regulation of phospho-EGFR, like described in a previous study [14].

The observed constitutive activation of Akt in our HNSCC cell lines was recently confirmed by Bussink et al. [39]. Additionally, clinical trials have shown a strong and independent association between activated Akt expression and treatment outcome [39]. Immediately after inhibition of the PI3K, we saw a down regulation of phospho-Akt, phospho-MEK and phospho-ERK on protein level, whereas phospho-MEK1/2 and phospho-ERK were up regulated through the lapse of the Akt dependent phosphorylation of Raf1 on Ser259 after 24 hours as shown by Zimmermann et al. [40].

Actually, the therapy of patients with HNSCC in the advanced stage III and IV implies primary radiotherapy in combination with a chemotherapy [5] and altered fractionation radiotherapy has a benefit for patient survival [31]. Our data indicate that a change in the therapeutic strategies of patients with HNSCC might be useful. Inhibition of the EGFR and/or downstream pathways in combination with the radiotherapy might be an option to the conventional radiation and chemotherapy of patients with HNSCC. In an animal model of nude mice it was shown, that the inhibition of the PI3K by LY294002 in combination with radiation induced a significantly better outcome [41]. Also in human studies involving HNSCC patients treated with a combination of radiation and EGFR antagonization an overall survival benefit was observed in 10%-15% of treated patients [42].

## Conclusion

Our results demonstrate that the EGFR and the downstream signals like PI3K/Akt and Raf/MEK/ERK are involved in radiation induced migration of HNSCC cells and might be a future target for the therapy of HNSCC in combination with radiotherapy.

## Competing interests

The authors declare that they have no competing interests.

## Authors' contributions

AP conceived of the study, performed the experiments, analysed the results and drafted the manuscript. JM carried out the irradiation experiments, the wound healing assay, the MTT assay and the immunoblot analysis. AK participated in the immunoblot analysis. TS participated in the wound healing assay. GP carried out and participated in the MTT assay, the immunoblot analysis and the modified boyden chamber assay. CB carried out the modified boyden chamber assay. ALB participated in the design of the study and performed the statistical analysis. EQS participated in the MTT assay. SP participated in the irradiation experiments. JS participated in the study design and the achievement of the assays. WA participated in the discussion of the results. RR conceived of the study, and participated in its design and coordination. All authors read and approved the final manuscript.

## Pre-publication history

The pre-publication history for this paper can be accessed here:

http://www.biomedcentral.com/1471-2407/11/388/prepub
